# Familial breast cancer: characteristics and outcome of BRCA 1–2 positive and negative cases

**DOI:** 10.1186/1471-2407-5-70

**Published:** 2005-07-04

**Authors:** Andrea Veronesi, Clelia de Giacomi, Maria D Magri, Davide Lombardi, Martina Zanetti, Cristina Scuderi, Riccardo Dolcetti, Alessandra Viel, Diana Crivellari, Ettore Bidoli, Mauro Boiocchi

**Affiliations:** 1 Division of Medical Oncology C, Centro di Riferimento Oncologico, Aviano, Italy; 2 Division of Experimental Oncology I, Centro di Riferimento Oncologico, Aviano, Italy; 3 Epidemiology Unit Centro di Riferimento Oncologico, Aviano, Italy

## Abstract

**Background:**

The clinical and pathological characteristics and the clinical course of patients with breast cancer and BRCA 1–2 mutation are poorly known.

**Methods:**

From 1997, patients with breast cancer and a family history of breast or ovarian cancer were offered BRCA testing. The clinical and pathological features of patients with known BRCA status were retrospectively assessed and comparisons were made between cancers arising in BRCA positive and BRCA wild type (WT) patients respectively. Type of treatment, pattern of relapse, event (local relapse, contralateral breast cancer, metastases) free and overall survival were also compared in the two groups. Out of the 210 patients tested, 125 had been treated and followed-up at our Institution and were evaluated in this study.

**Results:**

BRCA positive patients tended to be more often premenopausal (79% vs 65%) and to have positive lymphnodes (63% vs 49%), poorly differentiated tumours (76% vs 40% – p = 0.002 at univariate analysis, not significant at multivariate analysis) and negative estrogen receptors (43% vs 29%). Treatment was not different in the two groups. In the 86 BRCA-WT patients, the first event was a local relapse in 3 (3%), metachronous contralateral breast cancer in 7 (8%) and distant metastases in 16 (19%). In the 39 BRCA positive patients, the corresponding figures were 3 (8%), 8 (21%) and 3 (8%). There was no difference in event free survival, with a median of 180 months in both groups of patients. At 20 years, projected survival was 85% for BRCA positive patients and 55% for BRCA-WT, but this difference was not statistically significant.

**Conclusion:**

Although BRCA positive patients have more frequently negative prognostic factors, their prognosis appears to be equal to or better than in patients with BRCA-WT.

## Background

The issue of familial breast cancer has raised much attention in recent years due to its numerous medical and social implications. Following to the identification of the tumour suppressor genes BRCA1 and BRCA2 and of the increase in breast cancer risk associated with mutations of these genes, several studies have examined the entity of this risk and the diagnostic and therapeutic procedures indicated to curtail the incidence of breast and ovarian cancer in this population [1].

Whether the cases of breast cancer arising in BRCA1-2 positive women are different from those occurring in the general population in terms of biologic characteristics, response to treatment and eventual outcome has not been fully elucidated, although most reports suggest that BRCA1-2 positive cases differ from sporadic cases without familiarity for more frequent adverse prognostic factors [2].

At our Institution, a programme of identification of healthy BRCA1-2 carriers is ongoing, starting with the determination of the BRCA status in patients with breast cancer and a significant family history. This caused the identification of a population of breast cancer patients with family history, with or without BRCA1-2 mutation.

With the aim of ascertaining the differences in the clinical and pathological characteristics and in the clinical course of these two groups of patients, we retrospectively evaluated and compared several clinico-pathologic and therapeutic issues and the outcome of patients with or without BRCA1-2 mutation.

The results of that study are the subject of the present report.

## Methods

From 1997, patients with breast cancer presenting at our Institution were considered for eligibility to BRCA testing. Conditions of eligibility included a history among first degree relatives of: at least two cases of breast cancer <50 years; or at least 3 cases of breast cancer, any age; or at least two cases of bilateral breast cancer, any age; or at least two cases of ovarian cancer, any age; or at least one case of breast cancer <50 years plus one case of ovarian cancer, any age. Eligible patients were offered BRCA testing and consenting patients underwent genetic testing according to standard methods. In case of a positive test, genetic testing was also offered to healthy first degree relatives.

The clinical and pathological features of patients with known BRCA status were assessed and comparisons were made between cancers arising in BRCA positive and BRCA wild type (WT) patients respectively. Type of treatment, pattern of relapse, event (local relapse, contralateral breast cancer, metastases) free and overall survival were also compared in the two groups. In case of bilateral metachronous breast cancer, the primary tumour was considered to be the index cancer and the secondary tumour was classified as an event (contralateral breast cancer). Overall and progression free survival curves were estimated by the Kaplan-Meier technique and compared with the use of the two-sided log-rank test. The Cox proportional hazards model was fittted to assess the association with selected patient characteristics. The model included also terms for age (one-year age group) and tumour grade. A p value of less than 0.05 was required to reject the null hypothesis.

Out of the 210 patients tested, 125 had been treated and followed-up at our Institution and were evaluated in this study.

## Results

Of the 125 patients with known BRCA status followed or treated at our Institution, 86 were BRCA-WT and 39 were BRCA positive (9 BRCA1, 30 BRCA2). The main characteristics of the patients at the time of diagnosis of breast cancer and the primary treatment received are reported in Table 1. BRCA positive patients tended to be more often premenopausal (82% vs 64%) and to have positive lymphnodes (63% vs 49%), poorly differentiated tumours (76% vs 40% – p = 0.002 at univariate analysis, not significant at multivariate analysis)) and negative oestrogen receptors (43% vs 30%). BRCA-WT tended to be more often multifocal or multicentric (47% vs 33%). Only a minority of patients had a HER-2 determination. Previous treatment was not different in the two groups, although, as expected from oestrogen receptors distribution, more BRCA-WT patients received adjuvant hormone therapy and more BRCA positive patients received adjuvant chemotherapy. Although numbers were small, there was no significant difference between BRCA1 and BRCA2 cases. In the 30 BRCA2 cases, average age was 40.3 years, 62% of patients were premenopausal, 7% had T3-T4 tumours, 67% were node positive, 69% had G3 and 29% oestrogen receptor negative tumours.

The median follow-up time was 69 months (range, 10–280 months). In the 86 BRCA-WT patients, the first event was a local relapse in 3 (3%), metachronous contralateral breast cancer in 7 (8% of the 84 patients with primary unilateral breast cancer) and distant metastases in16 (19%). In the 39 BRCA positive patients, the corresponding figures were 3 (8%), 8 (21% of the 38 patients with primary unilateral breast cancer) and 3 (8%). The difference in the frequency of contralateral breast cancer as first event approached statistical significance (p = 0.07).

**Figure 1:**
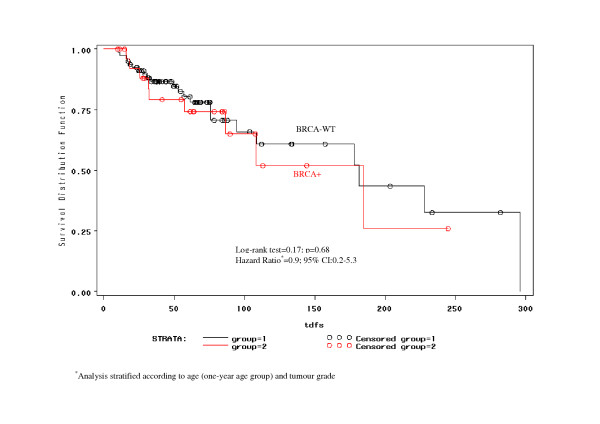
Event free survival.

**Figure 2:**
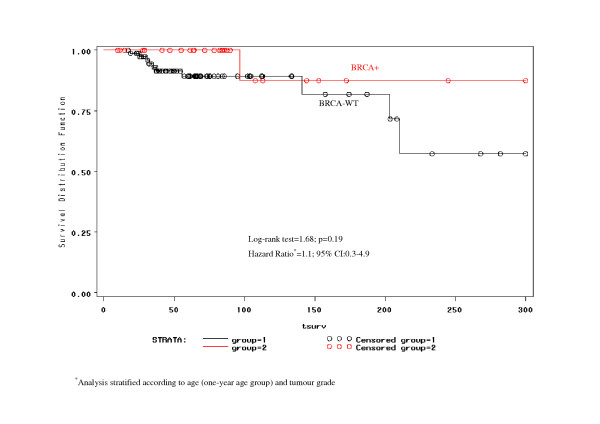
Overall survival.

Event free survival in BRCA-WT and BRCA positive patients is shown in Figure 1. There was no difference in event free survival, with a median of 180 months in both groups of patients. Overall survival is shown in Figure 2. At 20 years, projected survival was 85% for BRCA positive patients and 55% for BRCA-WT, but this difference was not statistically significant. There was no difference in event free survival and overall survival between BRCA1 and BRCA2 positive patients.

## Discussion

Although the importance of family history as a risk factor for breast cancer is widely recognized, there is disagreement on its impact upon prognosis, with conflicting results reported in several series [3,4]. The discovery of the breast cancer susceptibility genes BRCA1-2 has allowed a clearer identification of genetically related cases.

Some studies suggest that mutations of the BRCA gene may be related, besides their impact on the susceptibility to breast and ovarian cancer, to distinctive biological characteristics and clinical course.

In general, the histopathologic features of BRCA associated cases are reported as being more unfavourable as compared to sporadic cases, with more high-grade, oestrogen receptor negative and rapidly proliferating tumours [5]. In a recent study, nodal status was found to be correlated with tumour size in BRCA-1 negative but not in BRCA-1 positive patients [6]. In some relatively small studies, the prognosis of patients with BRCA mutations was worse [7-9] or comparable [10,11] to that of sporadic cases. In these series, prognostic factors were generally worse than in sporadic cases. In a Finnish study [12], it was noted that BRCA1 breast cancer patients had a lower survival rate than sporadic cases, BRCA2 patients or patients with non BRCA1/2 related familial breast cancer. However, the difference was not statistically significant. It should be noted that the BRCA positive patient populations studied varied much, including Ashkenazi Jewish patients with node negative disease [7], early-onset disease [8,9], or familial breast cancer [11,12]. Also control groups were different, varying from BRCA negative cases in the same population to large population based registries. The clinical implications of these findings appear not to be fully understood and more data on the issue are necessary before conclusions can be drawn. In particular, the impact of BRCA alterations upon survival is unclear [13].

In the present series, we analyzed the clinical characteristics, treatment and outcome in a group of patients with familial breast cancer according to their BRCA status.

The first finding to be mentioned is that, even in a group of breast cancer with a family history, the incidence of BRCA mutations is relatively low (about 25%) in our population of Italian women. This further stresses the need for stringent selection criteria before offering the test to the individual women. Besides, as a consequence, the study population was not large enough to detect small differences between BRCA positive and negative case..

The characteristics of patients differed in the two groups, in that features linked to an aggressive course (premenopausal status, poorly differentiated tumours, ER negative, node positive) were or tended to be more frequent in BRCA (predominantly BRCA2) positive patients. Treatment was similar.

The first event was more frequently distant metastases in BRCA-WT patients and contralateral breast cancer in BRCA positive patients. Time to progression was superimposable.

When survival curves are examined, one should take into account that the mechanism of the study caused long survivors to be overrepresented, which translates into very high long term survival rates (median survival has not been reached at 20 years). It appears that, while event free survival is superimposable, the percentage of long term survivors is higher among BRCA positive patients. This would be in accordance with a protective effect of BRCA, which counteracts the unfavourable prognostic factors associated with BRCA positivity. No difference emerged in our series between BRCA1 and BRCA2 positive patients, but the small number of BRCA1 cases in this predominantly BRCA2 series precludes conclusions. These findings are not entirely comparable with most data reported so far, in that in our series all patients had a family history of breast cancer and patients with BRCA-WT were probably positive for other unknown mutated genes. This is in agreement with a possible protective effect of BRCA positivity even within otherwise genetically related breast cancer.

## Conclusion

In conclusion, in this series it appeared that BRCA status interacted with known prognostic factors in determining the eventual outcome.

Whether BRCA may become a clinically useful prognostic or predictive factor can be ascertained only by large randomized trials. Prospective correlations within ongoing randomized studies of family history and BRCA status with outcome appear to be warranted.

## Competing interests

The author(s) declare that they have no competing interests.

## Authors' contributions

AV conceived the study and drafted the manuscript; CdG, MDM, DL and DC collected the data and were involved in the drafting and revision of the paper; RD and AV performed BRCA tests, collected the data and were involved in the drafting and revision of the paper; EB performed the statistical analysis; MB supervised laboratory work and was involved in the drafting and revision of the paper

## Pre-publication history

The pre-publication history for this paper can be accessed here:



## Figures and Tables

**Table 1:** Patient Characteristics and their corresponding hazard ratios*

CHARACTERISTICS OF PATIENTS	BRCA-WT (n = 86)	BRCA+ (n = 29)	p-value#	Hazard Ratio (95% CI)
Average age in years (range)	45.3 (22–82)	42.3 (26–62)	_	_
Laterality (right/left/bilat)+	35/41/5	15/13/1	0.68	1.3 (0.1–5.7)
Premenopausal	53/81 (65%)	23/29 (79%)	0.17	1.9 (0.5–6.7)
T3-T4	8/83 (10%)	3/23 (13%)	0.64	1.2 (0.3–6.5)
N+	37/76 (49%)	17/27 (63%)	0.20	1.4 (0.5–4.6)
G3	31/77 (40%)	19/25 (76%)	0.002	2.6 (0.8–7.2)
Multifocal-multicentric	38/82 (46%)	8/24 (33%)	0.26	0.8 (0.1–3.7)
ER-	20/70 (29%)	10/23 (43%)	0.18	1.5 (0.4–5.1)
Pgr-	22/72 (31%)	8/23 (35%)	0.71	1.1 (0.2–6.7)
HER2 2+/3+	11/31 (35%)	2/13 (15%)	0.18	0.6 (0.02–4.9)

*analyses stratified according to age (one-year age group) and tumour grade

# univariate chi-square

+ Hazard Ratio not computed for bilaterality

## References

[B1] Blackwood MA, Weber BL (1998). BRCA1 and BRCA2: from molecular genetics to clinical medicine. J Clin Oncol.

[B2] Chappuis PO, Nethercot V, Foulkes WD (2000). Clinico-pathological characteristics of BRCA-1 and BRCA-2 related breast cancer. Semin Surg Oncol.

[B3] Albano W, Recabaren J, Lynch H (1982). Natural history of hereditary cancer of the breast and colon. Cancer.

[B4] Andersson D, Badzioch M (1986). Survival in familial breast cancer patients. Cancer.

[B5] Chang J, Elledge RM (2001). Clinical management of women with genomic BRCA1 and BRCA2 mutations. Breast Cancer Res Treat.

[B6] Foulkes WD, Metcalfe K, Hanna W, Lynch HT, Ghadirian P, Tung N, Olopade O, Weber B, McLennan J, Olivotto IA, Sun P, Chappuis PO, Bégin LR, Brunet J-S, Narod SA (2003). Disruption of the expected positive correlation between breast tumor size and lymph node status in BRCA-1 related breast carcinoma. Cancer.

[B7] Foulkes WD, Chappuis PO, Wong N, Brunet JS, Vesprini D, Rozen F (2000). Primary node negative breast cancer in BRCA1 mutation carriers has a poor outcome. Ann Oncol.

[B8] Ansquer Y, Gautier C, Fourquet A, Asselain B, Stoppa-Lyonnet D (1998). Survival in early-onset BRCA1 breast cancer patients: Institute Curie Breast Cancer Group. Lancet.

[B9] Haffty BG, Harrold E, Khan AJ, Pathare P, Smith TE, Turner BC, Glazer PM, Ward B, Carter D, Matloff E, Bale AE, Alvarez-Franco M (2002). Outcome of conservatively managed early-onset breast cancer by BRCA 1/2 status. Lancet.

[B10] Johannsson OT, Ranstam J, Borg A, Olsson H (1998). Survival of BRCA1 breast and ovarian cancer patients: a population-based study from southern Sweden. J Clin Oncol.

[B11] Verhhoog LG, Brekelmans CTM, Seynaeve C, Dahmen G, van Geel AN, Bartels CC, Tilanus-Linthorst MM, Wagner A, Devilee P, Halley DJ, van den Ouweland AM, Meijers-Heijboer EJ, Klijn JG (1999). Survival in hereditary breast cancer associated with germline mutations of BRCA2. J Clin Oncol.

[B12] Eerola H, Vahteristo P, Sarantaus L, Kyyronen P, Pirhonen S, Blomqvist C, Pukkala E, Nevanlinna H, Sankila R (2001). Survival of breast cancer patients in BRCA1, BRCA2, and non-BRCA1/2 breast cancer families: a relative survival analysis from Finland. Int J Cancer.

[B13] Nicoletto MO, Donach M, De Nicolo A, Artioli G, Banna G, Monfardini S (2001). BRCA-1 and BRCA-2 mutations as prognostic factors in clinical practice and genetic counselling. Cancer Treatment Reviews.

